# Th17 suppression strategies: targeting IL-17 and RORγt pathways in autoimmune diseases

**DOI:** 10.3389/fimmu.2026.1941866

**Published:** 2026-10-27

**Authors:** Zeineb Zian, Mansur Aliyu, Fariha Hossain, Fatema Tuz Zohora, Ayça Ceylan, Nada Ben Moussa, Wan-Jen Hsieh, Feirouz Abbes, Sharafudeen D. Abubakar, Gholamreza Azizi

**Affiliations:** 1 Intelligent Automation and BioMed Genomics Laboratory (IABL), Faculty of Sciences and Techniques of Tangier, Abdelmalek Essaadi University, Tetouan, Morocco; 2 Department of Medical Microbiology and Parasitology, Faculty of Basic Clinical Science, College of Health Sciences, Bayero University, Kano, Nigeria; 3 Department of Pharmacy, Faculty of Health and Life Sciences, Daffodil International University, Savar, Bangladesh; 4 Jeffrey Cheah School of Medicine and Health Sciences, Monash University Malaysia, Subang Jaya, Selangor, Malaysia; 5 Department of Medical Biochemistry, Faculty of Medicine, Selcuk University, Konya, Türkiye; 6 Jefferson Headache Center, Department of Neurology, Thomas Jefferson University, Philadelphia, PA, United States; 7 LR21ES03 Research Laboratory “Oncogenesis and Tumor Progression”, Faculty of Medicine of Sousse, University of Sousse, Sousse, Tunisia; 8 Department of Medical Laboratory Science, College of Medical Sciences, Ahmadu Bello University, Zaria, Nigeria; 9 The Halvorsen Center for Computational Oncology, Memorial Sloan Kettering Cancer Center, New York, NY, United States; 10 Non-communicable Diseases Research Center, Alborz University of Medical Sciences, Karaj, Iran; 11 Department of Neurology, Thomas Jefferson University, Philadelphia, PA, United States

**Keywords:** autoimmune diseases, cytokines, IL-17, inflammation, plasticity, RORγt, Th17 cells

## Abstract

T helper 17 (Th17) cells and their key effector cytokine, particularly IL-17A and IL-17F, play central roles in host defense against extracellular pathogens. However, they are also critically involved in the pathogenesis of many autoimmune and inflammatory diseases. Th17 cell differentiation, stabilization, plasticity, and pathogenicity are regulated by a network of cytokines and transcription factors, including IL-6, TGF-β, IL-23, STAT3, and the key transcription factor RORγt. RORγt-dependent pathways are also relevant to other immune populations, including group 3 innate lymphoid cells (ILC3s), highlighting the complexity of the IL-17 axis across tissues. Dysregulation of these pathways can lead to chronic inflammation and tissue damage in diseases such as psoriasis, rheumatoid arthritis, multiple sclerosis, and inflammatory bowel disease. Therapeutic targeting of the Th17 axis has led to clinically established approaches, particularly direct blockade of IL-17 signaling and inhibition of IL-23, whereas direct RORγt inhibition remains an investigational strategy. RORγt inhibitors may modulate Th17 differentiation and broader RORγt-dependent transcriptional programs, but their clinical development has been challenged by variable efficacy, safety considerations, and translational limitations. This review provides a comprehensive overview of Th17 biology, focusing on differentiation, plasticity, tissue-specific functions, and the contribution of Th17 and other RORγt-expression populations to autoimmune diseases. Furthermore, it critically compares established IL-17-targeted therapies with emerging RORγt inhibitors, emphasizing their distinct mechanisms, cellular and molecular targets, clinical evidence, safety, and developmental status. Finally, we discuss combination strategies, translational challenges, and emerging biomarkers that may contribute to more individualized therapeutic approaches and patient stratification.

## 1 Introduction

T helper type 17 (Th17) cells play a crucial role in mucosal immunity and inflammation against extracellular bacterial and fungal infections. They achieve this by releasing cytokines such as IL-17 and IL-22, which stimulate inflammation. The differentiation of naive CD4+ T cells into Th17 cells is driven by the retinoic acid receptor-related orphan receptor gamma t (RORγt) transcription factor in response to specific cytokine signals such as IL-6 and IL-21 (1, 2). Th17 cells enhance barrier integrity and support antimicrobial immunity in the intestinal, lung, and skin mucosal surfaces. Moreover, they are involved in the recruitment, activation, and migration of neutrophils and other immune cells to sites of infection. Th17 cells contribute to inflammation by producing proinflammatory cytokines, including IL-17A, IL-17F, IL-21, and IL-22 (3). IL-17A and IL-17F target non-immune cells, including fibroblasts, endothelial cells, airway smooth muscle cells, and epithelial cells. They can also induce the expression of proinflammatory cytokines, such as IL-6, tumor necrosis factor (TNF)-α, and IL-1β (4). Th17 cells are a subpopulation of memory CD4+ T cells that express the C-C chemokine receptor type 6 (CCR6), which plays a role in humoral and cellular barrier defense (5). Th17 cells can be divided into four subclasses based on their chemokine receptor expression: Classical Th17 cells (CCR6+CCR4+CXCR3-), double-positive Th17 cells (CCR6+CCR4+CXCR3+), non-classical Th17.1 cells (CCR6+CCR4-CXCR3+), and double-negative Th17 cells (CCR6+CCR4-CXCR3-) (6). Classical Th17 cells are characterized by the production of high levels of IL-17A, whereas non-classical Th17.1 cells produce low levels of IL-17A and high levels of IFN-γ, and express T-bet. These cells have been found in peripheral blood and in inflammatory sites associated with many autoimmune diseases, including multiple sclerosis (MS), rheumatoid arthritis (RA), and inflammatory bowel disease (IBD) (7–9).

In healthy individuals, the balance of Th17 cell populations is regulated by mechanisms such as regulatory T cells (Tregs) and anti-inflammatory cytokines, including IL-10 (1). However, disruptions in the balance between Tregs and Th17 cells or overactivation of Th17 cells may contribute to the onset of autoimmune conditions and several inflammatory diseases, including asthma, RA, IBD, MS, psoriasis (Ps), systemic lupus erythematosus (SLE), and ankylosing spondylitis (AS) (10). Furthermore, innate lymphoid cell-3 (ILC3)s are innate immune cells that exhibit a cytokine profile similar to that of Th17 cells. They also respond to IL-23 and can rapidly produce IL-17 and IL-22 independently of antigen stimulation. Th17 cells arise from antigen-dependent adaptive immune responses and contribute to the persistence of chronic inflammation. By contrast, ILC3 cells respond rapidly to cytokine signals such as IL-23 and provide an antigen-independent source of IL-17 and IL-22 (11).

This review focuses on the key signaling pathways of Th17 cells, cellular plasticity and tissue-specific inflammatory responses as central therapeutic targets in autoimmune diseases. Furthermore, by presenting a new comparative framework that directly contrasts clinically validated downstream cytokine blockade with the emerging approach of upstream RORγt inhibition, it highlights the differences in their mechanisms of action and the levels of clinical evidence. A better understanding of these different points of intervention is of vital importance for improving therapeutic approaches, guiding research into the development of a new generation of medicines, and advancing personalized medicine in the treatment of autoimmune diseases.

A literature search was conducted and progressively updated during the preparation of the manuscript, with the final search performed in September 2026. The search was conducted across PubMed/MEDLINE, Web of Science, Scopus, and ScienceDirect using combinations of the following keywords: “Th17,” “IL-17,” “IL-17A,” “IL-17F,” “IL-17RA,” “RORγt,” “ILC3, “anti-IL17”, “Th17 suppression”, “RORγt inhibitors”, “autoimmune diseases”, “therapeutic targeting” “secukinumab,” “ixekizumab,” “brodalumab,” “bimekizumab,” “guselkumab,” and “risankizumab”. Evidence selection focused on peer-reviewed English-language studies addressing Th17 biology, RORγt-dependent transcriptional regulation, or therapeutic targeting of the IL-17/Th17 axis, including relevant preclinical *in vitro*/*in vivo* studies and Phase I–III clinical trials. Approved therapies, clinical-stage candidates, and preclinical compounds were selected based according to their relevance to the IL-17/Th17 axis and RORγt biology. Primary clinical studies, randomized controlled trials, and official regulatory or prescribing documents were prioritized over narrative reviews and case reports. Preclinical studies were included when they provided relevant mechanistic or pharmacological information not available from clinical evidence.

## 2 Th17 cell pathogenesis and its role in autoimmune diseases

### 2.1 Th17 cell pathogenesis

Dysregulation of cytokines, nutrients, or oxygen levels can trigger metabolic responses that result in Th17 plasticity and effector function (12, 13). Studies on Th17 cells have revealed their dual roles in health and disease. On one hand, they drive the pathogenesis of autoimmune diseases, while on the other, they help maintain immune equilibrium and tissue homeostasis. The ultimate role of Th17 cells depends on environmental cues, to promote a pro-inflammatory response against extracellular pathogens and mycotic agents or to contribute to inflammation through the expression of both anti-inflammatory and pro-inflammatory cytokines (**Figure 1**). Elucidating these mechanisms provides a basis for developing treatments for autoimmune diseases, allowing their immunomodulatory effects to be utilized without compromising host defense (14–16).

**Figure 1:**
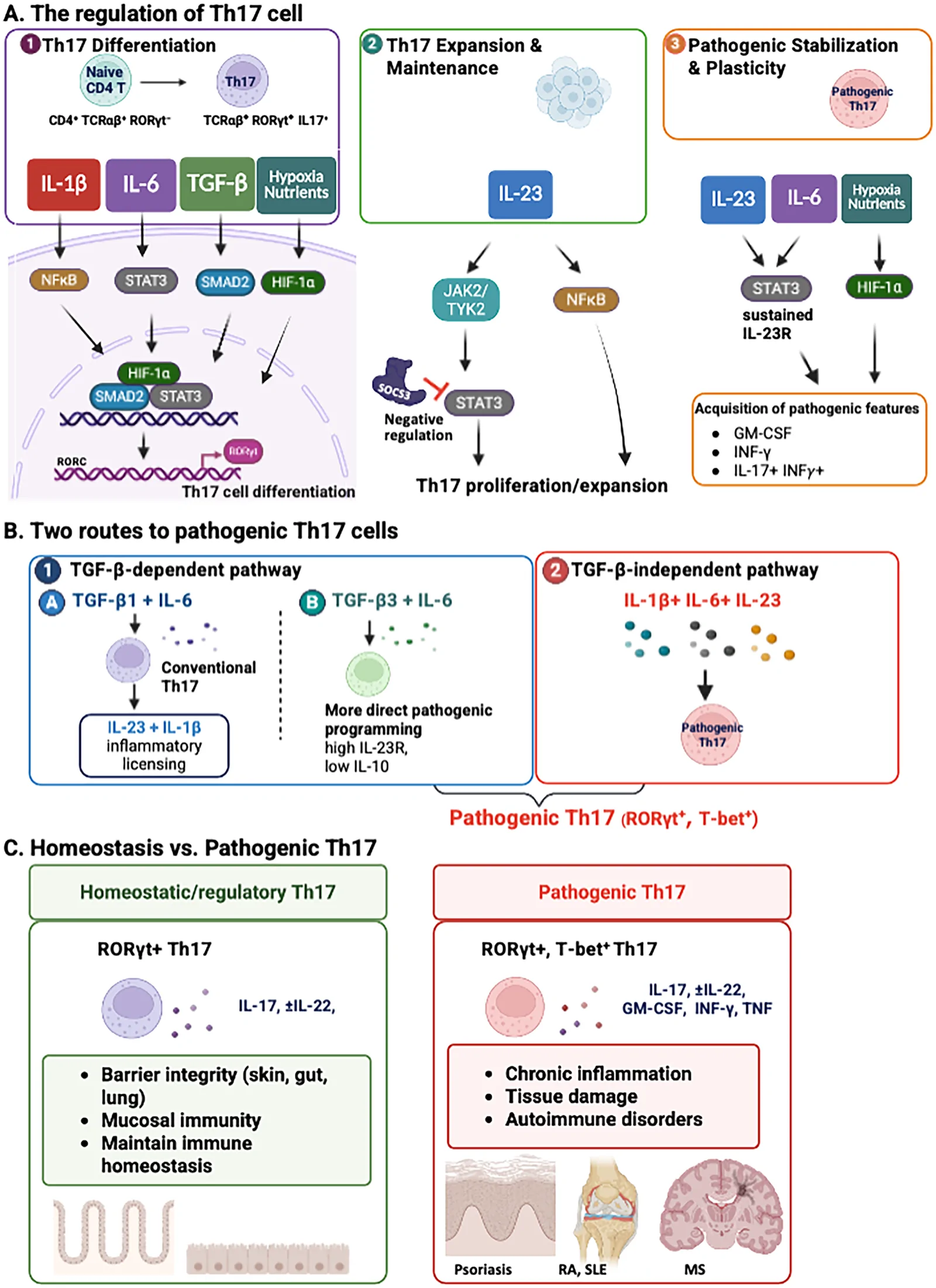
**(A)** Regulation of Th17 cells, illustrating three interconnected stages: (1) Th17 differentiation from naïve CD4+ T cells in response to cytokines and environmental signals, including IL-1β, IL-6, TGF-β, hypoxia, and nutrient availability; (2) IL-23-mediated Th17 expansion and maintenance through JAK2/TYK2 -STAT3 and NF-κB signaling; and (3) pathogenic stabilization and plasticity driven by sustained IL-23 signaling, IL-6, and metabolic cues, promoting the acquisition of pathogenic features, including GM-CSF, IFN-γ, and IL-17 production. **(B)** Two pathways leading to pathogenic Th17 cells: a TGF-β-dependent pathway involving TGF-β1 or TGF-β3 in combination with IL-6, and a TGF-β-independent pathway driven by IL-1β, IL-6, and IL-23. These pathways contribute to the acquisition of pathogenic Th17 characteristics. **(C)** Comparison of homeostatic/regulatory and pathogenic Th17 cells. Homeostatic/regulatory RORγt+ Th17 cells contribute to epithelial barrier integrity, mucosal immunity, and immune homeostasis, whereas pathogenic RORγt+ T-bet+ Th17 cells produce inflammatory mediators, including IL-17, IL-22, GM-CSF, IFN-γ, and TNF, and are associated with chronic inflammation, tissue damage, and autoimmune diseases, including psoriasis, RA, SLE, and MS.

Th17 cells exhibit plasticity, existing as functionally distinct states within the same lineage. Under inflammatory conditions driven by IL-23, IL-1β, and STAT3/STAT4 signaling, pathogenic Th17/Th1-like cells co-express RORγt, GM-CSF, IFN-γ, and tissue-homing receptors, driving chronic inflammation in the central nervous system (CNS), joints, and skin (7, 17–19). Although IL-23 is not required for initial differentiation, it stabilizes these disease-associated states, promoting expansion and pathogenicity (20–22). Conversely, in mucosal environments with TGF-β and retinoic acid, Th17-like cells may co-express RORγt and Foxp3, produce IL-10, and support barrier integrity and immune tolerance rather than tissue destruction. This tissue- and context-dependent balance explains why IL-17 pathway blockade is highly effective in psoriasis yet potentially harmful in active Crohn disease, where IL-17 and IL-22 contribute to mucosal defense and repair (21–23).

The IL-23/IL-17 axis is best viewed as a branching, dynamic network. IL-6, TGF-β, IL-1β, IL-21, and antigen-receptor signals can initiate or reinforce Th17-like programs. IL-23 promotes their persistence and pathogenic maturation. Tissue signals can also generate IL-23-independent IL-17 responses. This network includes conventional Th17 cells, pathogenic Th17/Th1-like cells, γδ T cells, MAIT cells, and ILC3s, with distinct developmental requirements and disease contributions (22). These distinctions justify comparing therapies. Anti-IL-17 biologics act downstream, neutralizing the effector pathway without directly reprogramming Th17 or ILC3 identity. RORγt inhibitors act upstream of several RORγt-dependent genes and may suppress multiple cytokine outputs and cell populations. However, this broader reach is not a proven clinical advantage and may interfere with protective IL-22 responses, thymic RORγt biology, and tissue homeostasis, goals incompletely achieved in humans (24, 25).

Th17 plasticity shapes treatment strategies because it determines which states and cytokine networks drive the disease at the time of treatment. If pathogenicity depends on IL-23-driven expansion and stabilization of Th17/Th1-like cells co-producing IL-17A, IFN-γ, and GM-CSF, IL-23 blockade may limit these populations, whereas IL-17A neutralization may suppress only part of the output while leaving IFN-γ- and GM-CSF-producing cells intact (21, 22). Conversely, ex-Th17 cells that have downregulated RORγt and IL-17A may become insensitive to RORγt inhibitors despite retaining an epigenetically imprinted Th17 origin and producing IFN-γ or inflammatory mediators (26, 27). This helps explain why some patients respond to IL-23 or IL-17 inhibitors, while others relapse or shift phenotypes (28, 29). Plasticity implies that one-cytokine suppression may not eliminate tissue-resident Th17-derived cells and may require the control of IL-1β, TNF, or tissue-specific signals to reprogram pathogenic circuits (22, 30).

Th17 biology is not exclusively IL-23-dependent. IL-17A/F can also be induced by T-cell-receptor stimulation, IL-1β, IL-6, TGF-β, microbial signals, and tissue-derived alarmins. IL-23-independent IL-17 responses may therefore occur in early or tissue-resident Th17-like cells and in non-conventional IL-17-producing γδT cells, MAIT cells, and ILC3s, depending on tissue and experimental context (31–33).

The differentiation and activation of Th17 cells are controlled by a complex network that includes the cytokine environment of IL-6, TGF-β, and IL-23, as well as transcription factors such as ROR-γt, STAT3, and BATF, along with epigenetic regulators (34, 35). Th17 cell differentiation and function are coordinated by interconnected signaling pathways that regulate their pro-inflammatory activity. TGF-β triggers signaling by binding to TGF-β receptor II (TGFβRII), which recruits and phosphorylates TGFβRI, leading to the activation of SMAD-dependent canonical pathways that primarily involve receptor-regulated SMADs 2 and 3. Once phosphorylated, these SMADs join with co-SMAD4 to form complexes that move into the nucleus to modulate gene expression. In Th17 differentiation, SMAD2 plays a predominant role by forming complexes with STAT3, which drives the expression of RORC and IL17 genes, contributing to Th17 lineage commitment (36). This function is in contrast with that of SMAD3, which promotes Treg cell differentiation. On the other hand, TGF-β also triggers SMAD-independent pathways, including MAPK, PI3K/AKT, and Rho-like GTPase signaling, which further modulate transcriptional networks and chromatin remodeling related to Th17 fate. Following the synthesis of the protein SMAD2, it may undergo post-translational modifications that regulate its intracellular localization and its interaction with STAT3. This regulation is crucial for modulating the TGF-β signaling pathway in Th17 cells (36, 37).

To stimulate Th17 cells, IL-23 is vital in the growth, survival, and pathogenicity of Th17 cells through activation of the STAT3 and nuclear factor kappa B (NF-κB) pathways. When IL-23 binds to the IL-23R on Th17 cells, it interacts with the JAK2 and tyrosine kinase 2 (TYK2), resulting in the phosphorylation and activation of STAT3. This promotes the expression of pro-inflammatory cytokines, including IL-17 and IL-22, and reinforces the Th17 transcriptional program (14). At the same time, IL-23 stimulates the NF-κB signaling pathway, which promotes inflammatory gene expression and enhances Th17 cell survival. When this pathway is dysregulated, particularly due to reduced SOCS3 expression, it amplifies Th17-mediated inflammation in diseases (Psoriasis (Ps) and MS). Thus, targeting the IL-23/STAT3/NF-κB axis may represent a promising therapeutic approach for Th17-mediated autoimmune disease (38).

Activated STAT3 and TGF-β-stimulated SMAD2 form complexes with each other to synergistically enhance the transcription of ROR-γt and other Th17-associated genes. Through the activation of STAT3, IL-23 signaling further amplifies the Th17 phenotype. Further signaling pathways contribute to Th17 differentiation and functional specialization in response to different cytokines and environmental factors (39). In addition, SMAD-independent mechanisms and adaptor proteins, such as SOCS proteins, present regulatory points that mediate immune activation and tolerance. This interaction of pathways can guide rational selection of therapeutic agents, including ROR-γt inhibitors and anti-IL-17 biologics, to block pathogenic Th17 responses while preserving host defenses (39, 40).

### 2.2 The role of Th17 cell in autoimmune diseases

Th17 cells contribute to the pathogenesis of various autoimmune diseases by producing specific cytokines, such as IL-17A, IL-17F, IL-22, and granulocyte-macrophage colony-stimulating factor (GM-CSF), as well as through interactions with resident tissue cells and other immune populations. The contribution of Th17 cells varies according to the disease and tissue context, involving distinct cytokine profiles and signaling pathways (14, 41). **Table 1** summarizes the reported roles of Th17 cells and the major cytokines and signaling pathways implicated in some autoimmune diseases.

**Table 1:** Role of Th17 in autoimmune diseases.

Disease	Th17 cell role	Key cytokines	Key mediators and signaling pathways	References
Psoriasis	Mediates skin inflammation, keratinocyte proliferation, and epidermal hyperplasia	IL-17A, IL-17F, IL-22, TNF-α, IL-21, IL-6	IL-23/IL-17 axis, ROR-γt, STAT3, NF-κB	(42–44)
Rheumatoid Arthritis	Contributes to synovial inflammation, cartilage destruction, and osteoclastogenesis	IL-17A, IL-17F, IL-22, TNF-α, IL-21, IL-6, GM-CSF	IL-6, IL-1β, IL-21/IL-23 axis, ROR-γt, STAT3, NF-κB, RANK/RANKL	(45, 46)
Multiple Sclerosis	Contributes to neuroinflammation, blood-brain barrier disruption, and demyelination	IL-17A, GM-CSF, IFN-γ, IL-21, IL-22	IL-23/IL-17 axis, ROR-γt, STAT3, JAK/STAT, NF-κB	(47–51)
Inflammatory Bowel Disease	Disrupts the intestinal barrier, recruits neutrophils, and enhances local inflammation	IL-17A, IL-21, IL-22, TNF-α	IL-23/IL-17 axis, STAT3	(52–56)
Systemic Lupus Erythematosus	Contributes to inflammation, tissue damage through autoantibody production, and cytokine release	IL-17A, IL-6, IL-21	IL-6/STAT3, IL-17 mediated feedback	(57–59)
Systemic Sclerosis	Involved in fibrosis and vascular inflammation	IL-17A, IL-6, TGF-β	TGF-β/SMAD, IL-17/NF-κB	(56, 60–63)
Ankylosing Spondylitis	Contributes to enthesitis, new bone formation, and local inflammation	IL-17A, IL-23, TNF-α	IL-23/IL-17 axis, NF-κB	(64–67)

## 3 Therapeutic targeting of Th17 pathways

IL-17 is a key pro-inflammatory cytokine produced by Th17 cells, playing a central role in coordinating immune responses and inflammatory processes in autoimmune diseases (68, 69). IL-17 drives inflammation by stimulating tissue-resident cells to release chemokines (CXCL1, CXCL2), granulocyte colony-stimulating factor (G-CSF), cytokines (IL-6, IL-8, and IL-1β, IL-19), and MMP, which promote neutrophil recruitment and activation, amplify immune cell infiltration, and facilitate tissue damage (70, 71). These factors reinforce mucosal barrier integrity and tissue repair while sustained IL-17 activity amplifies pro-inflammatory cascades, contributing to autoimmune disorders (72–74). Chronic overproduction of IL-17 leads to inflammation and tissue damage in Ps and RA, whereas short-term activity of IL-17 is essential for pathogen clearance and wound healing. This dual role of IL-17, both protective and pathogenic, makes it a critical target for managing autoimmune disease, highlighting the need for refined immunomodulatory approaches (75, 76). IL-17 cytokines produced by Th17 cells and ILC3s induce epithelial cells to produce antimicrobial peptides such as β-defensins and S100 proteins, which inhibit fungal growth (77, 78). Given the significant involvement of IL-17 in inflammation and the mechanisms of autoimmune diseases, therapeutic interventions targeting IL-17 signaling pathways use two main strategies, including direct neutralization of IL-17 cytokines and blockade of the IL-17 receptor complex (**Table 2**). Direct cytokine neutralization includes monoclonal antibodies (mAbs) targeting IL-17A, such as secukinumab and ixekizumab, which inhibit IL-17A binding to its receptor and blocking downstream inflammatory cascades (79, 80). Secukinumab and ixekizumab bind to IL-17A, which prevents receptor engagement and reduces neutrophil recruitment and inflammatory cytokine production in Ps and PsA. Receptor blockade targets the IL-17 receptor A (IL-17RA) via brodalumab, which binds to IL-17RA, inhibiting IL-17A, IL-17F, and their heterodimers, thereby blocking IL-17 signaling. This broader receptor-level blockade may be advantageous in settings involving multiple IL-17 family cytokines (75, 87, 88). This intervention reduces the inflammatory cascade that results in tissue damage in diseases such as Ps, AS, and PsA, leading to remission of skin lesions, reduced joint inflammation, and improved outcomes (75, 89).

**Table 2:** FDA-approved anti-IL-17 and IL-17RA inhibitors.

Drug name	Target/ mechanism	FDA-approved indications/clinical evidence (Response)	Dosing regimen	Common side effects	Notable warnings	References
Secukinumab	IL-17A Human IgG1	-Plaque psoriasis/PASI 75-81% -Psoriatic arthritis/ACR20 50-60% -Ankylosing spondylitis/ASAS20 60% -Non-radiographic axial spondyloarthritis, -Hidradenitis suppurativa/↓↓ abscess and inflammatory lesion	300 mg SC at weeks 0, 1, 2, 3, 4, then every 4 weeks (PsO); 150–300 mg every 4 weeks (PsA/AS)	Nasopharyngitis, upper respiratory infections, diarrhea, candidiasis	Caution in IBD	(79)
Ixekizumab	IL-17A Humanized IgG4	-Plaque psoriasis/PASI 75-90%, PASI 90-70% -Psoriatic arthritis/ACR20 -60% -Ankylosing spondylitis/ASAS20 -60% -Non-radiographic axial spondyloarthritis	160 mg SC at week 0, then 80 mg at weeks 2, 4, 6, 8, 10, 12, then every 4 weeks	Injection site reactions/pain, upper respiratory infections, nausea, tinea	Caution in IBD	(80, 81)
Brodalumab	IL-17RA Human IgG2	-Moderate-to-severe plaque psoriasis (for patients unresponsive to other systemic therapy)/PASI 75-80%, PASI 100-45%	210 mg SC at weeks 0, 1, 2, then every 2 weeks	Arthralgia, headache, fatigue, neutropenia, candidiasis, upper respiratory infections	Boxed warning for suicidal ideation/behavior, Contraindicated in active Crohn’s disease	(82–84)
Bimekizumab	IL-17A/IL-17F Humanized IgG1	-Moderate-to-severe plaque psoriasis/PASI 90-85%, PASI 100-60-70% -Psoriatic arthritis/Improve joint and spinal symptoms -Ankylosing spondylitis, -Non-radiographic axial spondyloarthritis, -Hidradenitis Suppurativa	320 mg SC (given as two 160 mg subcutaneous injections) during weeks 0, 4, 8, 12, 16, and every eight weeks thereafter (PsO/PsA with skin); 160mg every 4 weeks (AxSpA/PsA w/o skin)	Oral/genital candidiasis, upper respiratory infections, injection site reactions	Caution in IBD	(85, 86)

Randomized controlled trials have demonstrated that secukinumab, ixekizumab, and brodalumab rapidly induce remission of psoriatic plaques, improve PsA joint symptoms, and decrease radiographic progression in AS. Clinical response rates in Ps exceed 70% for skin clearance (Psoriasis Area and Severity Index 75 or higher), establishing IL-17 inhibitors as standard care (65, 90). In RA and related disorders, IL-17 contributes to inflammatory processes although clinical responses to IL-17 blockade have been variable and have not established this approach as a standard therapy (91). The outcomes vary compared to those of TNF or IL-6 biologics (88). In MS, IL-17 drives inflammation and lesion development. However, clinical trials have shown reduced MRI lesion activity and unclear effects on relapse rates, which require additional studies for therapeutic validation (92). Moreover, IL-17 inhibitors show limited benefit in MS and variable responses in RA, underscoring the need for complementary or combination approaches with established MS disease-modifying therapies (DMTs) (83, 87–89, 93–96).

### 3.1 Therapeutic targeting of Th17 pathways: anti-IL-17, anti-IL-23, and RORγt inhibitors

Therapeutic modulation of Th17 axis includes IL-17 blockade, upstream IL-23 inhibition, and direct RORγt targeting. Each approach offers distinct mechanistic advantages and challenges.

Anti-IL-17 therapies, such as secukinumab and ixekizumab, directly neutralize IL-17A cytokine activity, effectively interrupting downstream inflammatory cascades mediated by IL-17 receptor signaling. These agents demonstrate rapid clinical responses by suppressing effector cytokine production and tissue inflammation (97, 98). Anti-IL-23 agents (guselkumab and risankizumab) target the p19 subunit of IL-23, inhibiting the survival and expansion of pathogenic Th17 cells upstream. This strategy modulates Th17 cell maintenance without broadly suppressing innate IL-17 sources, potentially preserving host defense mechanisms better than direct IL-17 blockade (99, 100). RORγt inhibitors aim to suppress Th17 differentiation at the transcriptional level by inhibiting the master regulator RORγt, thereby reducing the expression of IL-17 and other pro-inflammatory genes. This upstream approach promises broader immunomodulation but faces challenges related to specificity and safety (35, 101).

### 3.2 Anti-IL-17/IL-17RA inhibitors: clinical evidence and safety

Multiple IL-17 inhibitors have been approved by the FDA and are widely used clinically for psoriasis, psoriatic arthritis, ankylosing spondylitis, and hidradenitis suppurativa. Secukinumab, ixekizumab, brodalumab, and bimekizumab exhibit robust efficacy with favorable safety profiles, though caution is advised regarding infection risk and, for brodalumab, psychiatric AE. These therapies have transformed the management of moderate-to-severe immune-mediated diseases, with high patient satisfaction and long-term durability (97, 102, 103).

Secukinumab (Cosentyx^®^), a fully human IgG1/κ mAb, was the first IL-17A inhibitor approved by the FDA in 2015 for moderate-to-severe plaque psoriasis, and later for PsA and AS (104, 105). It selectively binds to and neutralizes IL-17A and plays a crucial role in the pathogenesis of various immune-mediated diseases. By inhibiting the interaction of IL-17A with the IL-17R complex, secukinumab effectively blocks downstream signaling pathways, leading to reduced production of proinflammatory cytokines, such as TNF-α and IL-6. This suppression exhibits significant anti-inflammatory properties, especially in conditions such as psoriasis, PsA, and AS (106–108). Secukinumab reduces Ps features, such as keratinocyte hyperproliferation, acanthosis, and inflammation, which improves clinical outcomes. It modulates immunity by reducing neutrophil recruitment and disrupting the IL-17A-driven feedback loop. Common mild adverse events (AE) include upper respiratory tract infections and nasopharyngitis (75, 106, 109). Secukinumab, administered subcutaneously, reaches steady-state serum levels by week 16 and exhibits linear pharmacokinetics with a 27-day half-life and low immunogenicity. Gradual dose increases in some patients have made it possible to maintain efficacy at higher dosages. Secukinumab may alter CYP450 enzyme expression by reducing IL-17-mediated cytokine induction, which potentially affects drug metabolism (64, 106, 110).

Ixekizumab is a humanized IgG4 mAb with high affinity for IL-17A, targeting both the IL-17A homodimer and IL-17A/F heterodimer. This specific action disrupts IL-17A-driven inflammatory pathways in conditions such as moderate to severe plaque psoriasis, PsA, and axial spondyloarthritis (axSpA). Ixekizumab inhibits keratinocyte proliferation, decreasing the infiltration of inflammatory cells and normalizing cytokine profiles in affected tissues (111, 112). A study reported that Ixekizumab achieves peak plasma concentrations within 4 to 7 days following subcutaneous administration, with a half-life ranging from 14 to 18 days. The dosing regimen includes loading and maintenance phases that take place every two to four weeks (80). Ixekizumab shows high retention rates in long-term studies, exhibiting a safety profile characterized by mild to moderate AE, including infections and injection site reactions. Pooled analyses from randomized controlled trials support its long-term safety and no identified malignancy risk, although ongoing monitoring is recommended (19, 113). In a comparative study involving ixekizumab and secukinumab, ixekizumab showed a non-significant trend toward superior long-term efficacy and drug survival compared with secukinumab, although with slightly reduced tolerability (114). In another study, patients exhibited a favorable response to secukinumab, indicating its potential as an effective treatment option for those with an inadequate response or diminished efficacy to ixekizumab (115).

Bimekizumab is a humanized IgG1 mAb that targets both IL-17A and IL-17F. This dual inhibition provides broader suppression of IL-17 signaling than IL-17A-specific inhibitors (116–118). This disrupts IL-17A/F-mediated induction of inflammatory mediators such as CXCL1 and IL-6, downregulate *CXCL1, CXCL2, CXCL3 and IL-15RA*, reducing neutrophil infiltration and keratinocyte hyperproliferation (116, 119, 120). A large multicenter study among 834 patients reported that bimekizumab achieved PASI 100 response in 32.03% of patients within four weeks, increasing to 61.8% at 16 weeks and 78.92% by 36 weeks (121). Another study on 56 patients from two dermatology clinics with longer follow-up reported that 66% of the patients achieved PASI 100 at week 16 and 85.1% by week 52 (122). Bimekizumab has a half-life of 17–20 days with dosing every two to four weeks after the initial loading doses (123, 124).

Safety profiles from phase 2 and 3 trials showed tolerability consistent with IL-17 inhibition, with mucocutaneous candidiasis being the main AE (33). Disrupting the IL-17R-Act1-NF-κB signaling axis through therapeutic blockade causes impaired fungal clearance, leading to clinical candidiasis. These AE are direct and manageable consequences of the drug’s mechanism of action (108).

Brodalumab is a human IgG2 mAb targeting IL-17RA, thereby inhibiting multiple members of the IL-17 family (IL-17A, IL-17F, IL-17C, and IL-25). This action effectively disrupts IL-17-mediated inflammatory pathways (125). This receptor blockade prevents IL-17-induced activation of the ACT1 and NF-κB pathways, reducing keratinocyte proliferation and production of chemokines and cytokines, and suppressing chronic skin and joint inflammation (125). Unlike secukinumab and ixekizumab, which neutralize IL-17A, brodalumab inhibits the receptor for IL-17A and other cytokines, offering broader immunomodulation of Th17-driven inflammation (126). Bimekizumab has demonstrated high PASI 90 and 100 responses rates in clinical studies, although cross-trial comparisons with brodalumab should be interpreted cautiously because these data derive from different study population and trial designs.

Brodalumab is administered subcutaneously at a dose of 210 mg every two weeks after an initial loading regimen. In the phase III AMAGINE-2 and AMAGINE-3 trials, brodalumab 210 mg achieved PASI 75 responses of 86.3% and 85.1%, respectively, at week 12, while PASI 100 responses were 44.4% and 37%, respectively (127). The phase III AMAGINE studies demonstrated the rapid therapeutic effects of brodalumab. At week 12, brodalumab 210 mg produced significantly higher PASI 75 responses than placebo (86% vs. 8% in AMAGINE-2 and 85% vs. 6% in AMAGINE-3) and higher PASI 100 responses than ustekinumab (44% vs. 22% and 37% vs. 19%, respectively) (127).

Studies have confirmed its effectiveness and safety, with the most frequently reported AE being mild infections such as candidiasis and nasopharyngitis (113). Brodalumab provides rapid improvement even in patients with multiple risk factors or those who have not responded to previous biologic treatments (128). It includes a boxed warning regarding the risk of suicidal thoughts, requiring psychiatric follow-up, although causality remains to be confirmed (113). The efficacy of brodalumab in treating IL-17-driven diseases has been established (129), demonstrating high rates of complete clearance in Plaque Psoriasis, particularly among patients resistant to biologics (123). Clinical data have shown promising results for severe forms, such as pustular psoriasis, suggesting effective inhibition of the IL-17 pathway in reducing neutrophilic inflammation (84). The AMVISION studies confirm the effectiveness of brodalumab in PsA, showing improvements in joint symptoms and disease activity (130).

### 3.3 RORγt inhibitors: development, efficacy and translational limitations

RORγt inhibitors, including antagonists and inverse agonists, primarily target the ligand-binding domain (LBD) of RORγt and interfere with its active transcriptional function. This leads to the stabilization of inactive conformations and prevents the recruitment of coactivators, which results in the suppression of IL-17 and related cytokines (101, 131, 132). Animal models of psoriasis, experimental autoimmune encephalomyelitis (EAE), and type 1 diabetes consistently show reduced disease severity, decreased tissue infiltration of Th17 cells, and improved clinical and histopathological scores upon RORγt blockade. Notably, topical and oral formulations have demonstrated appreciable efficacy and tissue selectivity, minimizing systemic exposure (133). Multiple RORγt inhibitors have been evaluated across preclinical and early clinical development programs (**Table 3**). Compounds such as JNJ-61803534 and IMU-935 are significant for target engagement and suppression of IL-17A release in human cellular assays. Single ascending dose trials have shown these compounds to be well-tolerated and to have robust pharmacodynamic effects (134, 146). Nevertheless, translation to human efficacy has been challenging, with several agents failing in clinical trials due to limited benefit or safety concerns despite maintained pharmacological activity and promising preclinical outcomes (133, 134).

**Table 3:** Pharmacological characteristics, clinical and development, efficacy, and safety of selected RORγt inhibitors.

Compound and developer	Pharmacology and binding mode	Selectivity and reported result	Potency assay and reported result	Route; indication; trial ID; highest phase	Current status and principal efficacy/safety finding	References
JNJ-61803534; Janssen Research & Development	**RORγt inverse agonist**; ligand-binding-domain modulator. The precise orthosteric/allosteric designation is not established consistently in the clinical publication; it should not be labeled definitively without the medicinal-chemistry paper.	Selective for **RORγt over RORα/RORβ**; also tested against other nuclear receptors.	RORγt-driven transcriptional reporter and human CD4^+^ Th17 differentiation assays; inhibited IL-17A but not Th1 IFN-γ or Treg differentiation. The published article emphasizes functional potency but does not provide a universally transferable single IC_50_ across all assays.	Oral; psoriasis was the proposed indication. **NCT03139500**; phase 1.	**Terminated.** The phase-1 study reported acceptable short-term clinical safety, pharmacokinetic/pharmacodynamic activity, and no clinically important thymic signal; development was subsequently discontinued after embryo–fetal toxicity findings in rabbits, as discussed in the published development account.	(97, 131, 134)
JNJ-54271074; Janssen	**RORγt inverse agonist**; reported as a ligand-binding-domain modulator. Orthosteric versus allosteric binding is not established in the cited peer-reviewed source.	Reported selective for **RORγt over RORα and RORβ**.	RORγt transcriptional assay: **IC_50_ ≈9 nM**; human IL-17A cellular assay: **IC_50_ ≈17 nM**. Oral dosing reduced collagen-induced arthritis in mice.	Oral in animal studies; autoimmune inflammation/arthritis was the experimental indication. No verified human trial identifier; no clinical phase established.	**Preclinical/discovery status.** Efficacy was demonstrated in a collagen-induced arthritis model; a published human safety, efficacy, or termination dataset was not identified.	(32, 100, 131)
SR2211; Scripps Research/academic collaborators	**RORγ-selective inverse agonist**; ligand-binding-domain modulator. The original report supports ligand-dependent receptor modulation but does not establish a clinically relevant orthosteric/allosteric classification.	Highly selective for **RORγ over RORα**; no detectable RORα binding in the reported assays.	Radioligand binding: **Ki=105K_i=105Ki​=105 nM**; Gal4–UAS luciferase transcription assay: **IC_50_≈320 nM**; also suppressed *Il17a*, *Il23r*, and Th17 differentiation in cellular assays.	No clinical route; experimental autoimmune models included intraperitoneal dosing in mice. No clinical indication or trial ID.	**Experimental/preclinical tool compound.** It reduced Th17 responses and arthritis in mice, but no human development or human safety evidence has been reported. [3,4]	(131, 135)
IMU-935; Immunic Therapeutics	**RORγ/RORγt inverse agonist**; reported to disrupt RORγt transcriptional activity and RORγt–CBFβ interaction. The public peer-reviewed record does not justify a definitive orthosteric/allosteric label.	Designed to inhibit **RORγ/RORγt**; comparative selectivity data should be taken from the sponsor’s assay package rather than inferred from the clinical literature.	Potency has been reported in company clinical-development materials and conference data; a definitive, independently published, assay-normalized IC_50_ with biological matrix is not consistently available.	Oral; studied in psoriasis and oncology. **NCT038 dairy?** The verified oncology study is **NCT05124795**; phase 1.	**Clinical-stage, with indication-specific development.** Early phase-1 data supported tolerability and pharmacodynamic activity; the published prostate-cancer phase-1 report found no dose-limiting toxicities but no robust antitumor activity in an unselected cohort.	(136, 137)
Allosteric compounds 1–3; academic medicinal-chemistry programs	**Allosteric RORγt modulators** where the cited structural work supports occupation of the noncanonical allosteric pocket. Classification as inverse agonist depends on the individual compound and assay.	Usually evaluated for RORγt selectivity over RORα/RORβ; compound-specific data must be reported separately.	RORγt ligand-binding, co-regulator recruitment, reporter, or structural assays; no single validated potency value can be assigned to the aggregate label “Cpd 1–3.”	No clinical route, indication, or trial ID.	**Preclinical/experimental.** These are discovery-stage probes; published studies establish mechanism and structure–activity relationships, not clinical efficacy or safety.	(138, 139)
Triazine derivatives; academic medicinal-chemistry program	**RORγt inverse agonists**; binding mode is ligand-binding-domain modulation, but orthosteric versus allosteric assignment should be stated only for the individual co-crystallized compound.	Reported as RORγt-focused compounds; isoform selectivity varies by analog.	Dual-FRET biochemical assay: approximately **7–50 nM** for the series; compound 14g: **22.9 nM**. Cell reporter assay for 14g: **0.428 μM**.	No human route, indication, or trial ID.	**Preclinical/experimental.** The series suppressed RORγt transcription in biochemical and cellular assays; no animal or human clinical efficacy/safety program was established in the cited article.	(140, 141)
Cpd 1, imidazopyridine/triazolopyridine-derived series; academic medicinal-chemistry program	**RORγt inverse agonist**; ligand-binding-domain modulator. For individual analogs, structural data support binding within the RORγt ligand-binding pocket; the precise orthosteric/allosteric nomenclature should follow the relevant co-crystal structure.	Developed for RORγt selectivity; compound-level RORα/RORβ data should be cited separately.	Biochemical RORγt and cell-based reporter assays; optimized analogs showed strong inhibition and oral PK/PD activity in rodents, including reduction of IL-17 production after oral dosing. [9,10]	Oral gavage in rodent PK/PD studies; autoimmune disease was a proposed indication. No human trial ID or clinical phase.	**Preclinical/experimental medicinal chemistry.** The principal result was oral exposure with pharmacodynamic IL-17 suppression in rodents; human efficacy and safety are unavailable.	(142–145)

Bold values indicate the principal reported potency (IC50 or Ki) for each compound. Assay type is specified for each value; cross-assay comparisons should be made with caution. RORγt, retinoic acid-related orphan receptor gamma t; IC50, half-maximal inhibitory concentration; Ki, inhibition constant; PK/PD, pharmacokinetic/pharmacodynamic; NCT, National Clinical Trial identifier.

Although RORγt inhibitors exhibit strong selectivity and potential for disease modulation, safety remains a considerable challenge in their clinical development. There are concerns regarding on-target effects affecting thymocyte maturation and viability, which have resulted in thymic hyperplasia and lymphoma in rodent models due to the involvement of RORγt in thymocyte survival. Even with efforts to distinguish efficacy from the risk of lymphoma, developing compounds that effectively balance IL-17 inhibition with thymocyte apoptosis and can be translated into clinical practice remain challenging (134). Some inhibitors, such as IMU-935 and BMS-986251, selectively suppress Th17 differentiation without impeding thymocyte survival. However, long-term safety data remain limited, and an incomplete understanding of the relevance of rodent lymphomagenesis in humans hinders progress. Additional risks include systemic toxicities, off-target effects, microbiota interactions, and uncertainty regarding optimal inhibition levels for efficacy without impairing protective immunity (146, 147).

Comparatively, anti-IL-17 therapies were well used in clinical studies, compared to RORγt inhibitors, with many of them approved for the treatment of patients by the relevant organizations.

RORγt inhibitors remain predominantly in preclinical and early clinical development. Compounds such as JNJ-61803534 and IMU-935 have advanced to phase 1/2 trials. However, they face hurdles in achieving therapeutic windows without AE. Most RORγt inhibitors, including SR2211 and novel covalent or allosteric modulators, are still experimental and are optimized for potency and selectivity in cellular and animal models (35, 138, 140, 148).

Several RORγt inhibitors have been discontinued or halted because of limited efficacy, safety concerns, or unfavorable pharmacokinetics. However, key limitations include insufficient selectivity, leading to off-target effects, poor oral bioavailability, and metabolic instability. Additionally, some compounds failed to demonstrate adequate clinical benefits over existing biologics in early trials (35, 149). A critical safety concern is thymic toxicity observed in preclinical species. RORγt is essential for thymocyte development and lymphoid tissue organization. Its inhibition can cause thymic atrophy and altered T cell maturation in rodents and non-human primates. However, this risk has tempered enthusiasm for systemic RORγt inhibition and prompted efforts to develop tissue-selective or peripherally restricted inhibitors (103, 150, 151).

Preclinical studies have demonstrated that RORγt antagonism induces thymic involution and disrupts γδ T cell and innate lymphoid cell development in mice, raising concerns about immunosuppression and infection susceptibility. The clinical relevance of these preclinical thymic effects remains uncertain. Although early clinical studies have not established a clear thymic toxicity signal, the limited number of exposed patients and duration of follow-up preclude definitive conclusions regarding long-term risk. Ongoing monitoring and biomarker studies are essential for fully assessing long-term safety (150–153).

### 3.4 Anti-IL-23

Guselkumab and risankizumab are monoclonal antibodies that selectively target the p19 subunit of IL-23, a pivotal cytokine driving the differentiation and maintenance of Th17 cells implicated in inflammatory diseases. IL-23 is a heterodimeric cytokine composed of a p19 subunit exclusive to IL-23 and a p40 subunit shared with IL-12, and it acts as a key upstream regulator of the IL-23/Th17 axis. By antagonizing IL-23, these agents disrupt the JAK2–TYK2–STAT3–dependent expansion, maintenance, and pathogenic activity of Th17 cells, reducing downstream production of IL-17A, IL-17F, IL-22, and TNF-α that drive keratinocyte hyperproliferation and chronic inflammation in psoriasis (154–156).

IL-23’s role in promoting the pathogenic Th17 response underlies conditions such as psoriasis, psoriatic arthritis, and Crohn’s disease. Clinical trials demonstrate that guselkumab effectively induces and maintains remission in moderate-to-severe plaque psoriasis and psoriatic arthritis, with favorable safety profiles observed in long-term studies (157, 158). In Crohn’s disease, risankizumab induces clinical remission in up to 45% of patients, and guselkumab is emerging as a promising treatment option with ongoing phase III trial success (159, 160).

In the VOYAGE 1 and VOYAGE 2 phase III trials, guselkumab (100 mg at weeks 0 and 4, then every 8 weeks) achieved PASI 90 in approximately 70–73% and PASI 100 in about 29–37% of patients by week 16, superior to placebo and adalimumab. In the UltIMMa-1 and UltIMMa-2 trials, risankizumab (150 mg at weeks 0 and 4, then every 12 weeks) induced PASI 90 in roughly 71–75% and PASI 100 in 36–51% of patients at week 16, outperforming ustekinumab. Together, these clinical data support selective IL-23p19 inhibition as an effective therapeutic strategy for targeting the IL-23/Th17 axis in moderate-to-severe plaque psoriasis (161–163).

## 4 Comparative analysis between anti-IL-17 and RORγt Inhibition

Dysregulated IL-23/IL-17 signaling represents a central driver across multiple autoimmune and chronic inflammatory pathologies (164). The therapeutic manipulation of the IL-23/IL-17/Th17 axis involves two mechanistically distinct strategies. The first strategy entails the extracellular neutralization of IL-17-family cytokines or their receptor signaling using monoclonal antibodies, a method that has been clinically validated in several inflammatory diseases. The second strategy focuses on the intracellular inhibition of the Th17 transcriptional network through RORγt inverse agonists or antagonists, which is primarily an investigational approach with oral administration (22).

RORγt inhibitors act upstream at the nuclear transcription factor level, whereas anti-IL-17 biologics act downstream by neutralizing IL-17A, IL-17F, or IL-17 receptor signaling. RORγt inhibition can reduce a coordinated Th17 transcriptional program, including IL-17A, IL-17F, IL-22, IL-23R, and CCR6, and can affect conventional Th17 cells as well as RORγt-dependent γδ T, NKT, and ILC3 populations (32, 131, 134). However, this broader molecular and cellular reach is a pharmacological property, not a proven clinical advantage, over selective IL-17 blockade. The two strategies were compared below (**Table 4**).

**Table 4:** Anti-IL-17 monoclonal antibodies vs small molecule RORγt inhibitors/inverse agonists.

Parameter	Anti-IL-17 monoclonal antibodies (e.g., secukinumab, ixekizumab, bimekizumab, brodalumab)	Small molecule RORγt inhibitors/inverse agonists (e.g., IMU-935, AZD0284, TMP778, VTP-43742)	References
Primary Molecular Target	Soluble IL-17A, IL-17F, or cell-surface IL-17RA subunit	Nuclear receptor transcription factor RORγt (RORC2 isoform)	(131)
Position in Signaling Axis	Distal effector level (neutralizes released cytokines or blocks extracellular receptor)	Upstream master regulator level (inhibits DNA transcription within target cells)	(131)
Cytokine Profile Suppressed	Narrow: IL-17A (secukinumab, ixekizumab), IL-17A/F (bimekizumab), or IL-17A/F/E/C signaling (brodalumab)	Broad: *IL17A*, *IL17F*, *IL22*, *IL26*, *IL23R*, *CCR6* transcription	(131)
Cell Populations Impacted	Indirect effector blockade; no direct alteration of producing cell lineage integrity	Direct modulation of Th17, Tc17, ILC3s, γδ-T17, and DP thymocytes	(165)
Impact on Th17/Treg Plasticity	Negligible direct effect on T cell transcriptional fate or Foxp3 expression	Suppresses Th17 commitment; can favor Foxp3^+^ Treg skewing or lineage deviation	(98)
Route & Pharmacokinetics	Subcutaneous/intravenous injection; long half-life (~20–28 days); intermittent dosing	Oral administration; short elimination half-life; requires daily dosing	(80, 124, 134, 136)
Safety & Toxicity Concerns	Mucocutaneous candidiasis; neutropenia; inflammatory bowel disease concerns vary by individual agent	Hepatotoxicity, elevated transaminases, thymocyte apoptosis, theoretical oncogenesis	(166, 167)
Development Status	Approved biologic therapies in multiple rheumatologic and dermatologic indications	Experimental; Frontrunner candidates discontinued in Phase I/II due to toxicity or efficacy ceilings	(168, 169)

In RA models, JNJ-54271074 inhibited RORγt-dependent differentiation and reduced IL-17A production from memory T cells, as well as IL-17A- and IL-22-producing γδ T and NKT cells. Oral treatment attenuated collagen-induced arthritis, whereas ex vivo samples from patients with RA showed reduced IL-17A responses (32). JNJ-61803534 also inhibited RORγt-driven IL-17A, IL-17F, IL-22, and IL-23R expression and reduced arthritis in mice. It also inhibited IL-17A/IL-22-producing γδ T cells in an imiquimod-induced skin inflammation model (134). These findings support broader target engagement across RORγt-dependent populations but do not establish superior efficacy in human RA. Indeed, anti-IL-17 agents have shown only modest and heterogeneous benefits in RA, and the available clinical evidence does not demonstrate that upstream RORγt inhibition overcomes the dominant contributions of TNF, IL-6, B cells, and myeloid inflammation (170–173).

The same distinction applies to Th17 plasticity. Anti-IL-17 antibodies neutralize extracellular cytokine activity without directly affecting the differentiation, survival, or transcriptional identity of cytokine-producing cells. RORγt inhibitors can interfere with Th17 commitment and maintenance and may influence Th17/Th1 or “ex-Th17” states, in which pathogenic cells acquire T-bet and IFN-γ expression levels. Nevertheless, RORγt inhibition should not be described as selectively converting Th17 cells into regulatory T cells in humans; such lineage effects remain context-dependent and are supported mainly by experimental studies. Moreover, suppressing IL-22 and IL-17 may be beneficial in some inflammatory tissues but harmful in others because IL-22 contributes to epithelial repair and mucosal defense (134).

Disease-specific efficacy further emphasizes the difference between mechanisms and clinical validation. Anti-IL-17A agents such as secukinumab and ixekizumab, and dual IL-17A/F inhibition with bimekizumab, have reproducibly demonstrated high efficacy in psoriasis and psoriatic arthritis, with many trials reporting PASI75 responses of approximately 80-90% and substantial PASI90/100 responses (174–176). Their efficacy has also been established in axial spondyloarthritis. In contrast, JNJ-54271074, JNJ-61803534, FC99, W14, and related compounds have shown activity in psoriasis, arthritis, and experimental autoimmune encephalomyelitis-type (EAE)-type models. However, these data remain preclinical (32, 131, 134, 177). Secukinumab produced a signal of reduced MRI lesion activity in a small proof-of-concept multiple-sclerosis study, although the primary MRI endpoint was not statistically significant. This does not constitute an approved or established MS indication (170, 172, 173).

This distinction is particularly important in Crohn’s disease. IL-17 blockade failed to provide benefits and was associated with disease-worsening signals in clinical studies, consistent with the role of IL-17 in intestinal barrier protection. BI119 reduced Crohn’s disease-associated Th17 signatures and experimental colitis while preserving mucosa-associated IL-22 responses in ex vivo and translational models (170). This finding demonstrates a potentially useful context-specific design principle, selective suppression of pathogenic RORγt outputs while preserving protective IL-22. However, it does not show that systemic RORγt inhibition is clinically safer or more effective than anti-IL-17 therapy in Crohn’s. Therefore, it should be presented as a translational proof-of-concept rather than a therapeutic advantage.

The safety profiles of these agents reflects their pharmacological positions. Anti-IL-17 biologics have extensive long-term human clinical trial and post-marketing safety experience, with a relatively defined mechanism-related profile that includes mucocutaneous candidiasis, upper respiratory infections, uncommon neutropenia, and rare new-onset or exacerbated inflammatory bowel disease. Although these risks are generally infrequent, they remain clinically relevant because IL-17 signaling contributes to mucosal antifungal defense and intestinal barrier homeostasis (65, 178, 179).

The safety findings of RORγt inhibitors were compound-specific. Early systemic programs reported dose-related transaminase elevations, while JNJ-61803534 was constrained by embryo-fetal developmental toxicity in non-clinical studies, despite generally acceptable Phase I human tolerability. RORγ-dependent thymocyte survival raises concerns regarding thymocyte apoptosis and long-term thymic toxicity, and RORγ-deficient rodents develop thymic lymphomas. However, these animal findings do not establish human oncogenicity. Mild gastrointestinal symptoms and fatigue have occurred with selected compounds but should not be generalized as class effects. Overall, clinical attrition has reflected a combination of safety findings, non-clinical toxicology, inadequate efficacy, and failure to achieve a sufficiently competitive therapeutic margin (108, 134, 136, 180).

RORγt inhibition has a broader mechanistic reach than IL-17A neutralization because it can repress multiple RORγt-dependent genes and modulate several IL-17-producing cellular populations. Whether this broader target engagement improves clinical efficacy or immune system balance remains unproven and may involve trade-offs, including suppression of protective IL-22 biology, thymic toxicity, hepatic liabilities, and uncertain long-term effects. In contrast, anti-IL-17 biologics provide a narrower, downstream pathway blockade supported by substantially stronger evidence of human efficacy and safety (142).

Currently, anti-IL-17 therapies are established clinical treatments for psoriasis, psoriatic arthritis, and axial spondyloarthritis, whereas RORγt inhibitors remain investigational. JNJ-61803534 reached early clinical evaluation and demonstrated pharmacodynamic activity. However, available evidence does not support positioning it as a clinically superior alternative to IL-17 biologics. Although VTP-43742 provided early proof-of-concept in psoriasis, it encountered development concerns, including reversible transaminase elevation at higher exposure. Newer inverse agonists and tissue-restricted compounds may improve the therapeutic index. However, their status should be described as exploratory until adequately powered randomized trials establish their comparative efficacy, organ safety, infection risk, and durability (181).

## 5 Emerging therapies and future directions

Several preclinical studies have demonstrated potential efficacy of RORγt inhibition in treating systemic sclerosis, RA, and Ps (142, 148, 182). A novel RORγt inhibitor was tested in a Phase II clinical trial for moderate to severe Plaque psoriasis. The BI 730357 showed only moderate efficacy compared to the placebo and was well-tolerated. Other RORγt inhibitors, such as PF-06763809 and ABBV-553, were tested in clinical trials and resulted in less efficacy and safety in Ps patients, which led to the suspension of these clinical trials (183). These findings have prompted interest in exploring whether RORγt inhibitors could be combined with other targeted therapies to improve efficacy or therapeutic index. However, clinical evidence supporting such combinations is currently lacking. To date, no study has specifically evaluated RORγt inhibitors combined with other therapeutic agents in targeting the IL-17/Th17 axis within autoimmune diseases.

IL-17 inhibitor monotherapy showed effective results in treating patients with Ps, PsA, or axSpA (81, 113, 184, 185). However, this led to limited efficacy and several AE in other autoimmune diseases such as IBD (186), RA (Pavelka et al., 2015), and Ps (187). These contradictory results have prompted the development of IL-17 inhibitors combined with other therapeutic agents to improve the treatment response in different autoimmune diseases. Anti-IL-17 agents combined with other cytokine-targeted therapies, such as anti-TNF and anti-IL-1β agents, were tested in ex vivo as well as *in vivo* models. The synergic effect of anti-IL-17/anti-TNFα antibodies in cultures of human fibroblast-like synoviocytes (FLS) co-stimulated with IL-17 and TNFα showed the significant inhibition of cytokine, chemokine, and matrix enzyme responses (188). Likewise, compared to monotherapy, anti-IL-17 combined with anti-TNFα or anti-IL-1β has successfully reduced the disease severity in a collagen-induced arthritis mouse model (189) (190). However, some clinical studies reported limited efficacy of dual inhibition of IL-17 and TNF- α. In randomized controlled trials, the bispecific antibody ABT-122 exhibited a similar efficacy and safety profile compared to adalimumab in PsA 1 (130) and RA patients 2 (191). Similarly, in a 24-week open-label extension of 12-week randomized, double-blind studies, ABT-122 exhibited maintained efficacy and mild or moderate AEs for up to 36 weeks in patients with RA or PsA on background MTX 3 (192). Overall, available clinical evidence has not demonstrated a clear efficacy or safety advantage of combined IL-17 and TNF-α blockade over established single-target approaches.

### 5.1 Alternative approaches for Th17 suppression

Previous studies in animal models as well as clinical trials revealed that GM-CSF inhibition is significantly effective, which indicates that GM-CSF plays a critical role in the pathogenesis of Th17 cells and autoimmune development (193). In this context, researchers combined anti-IL-17 with GM-CSF to test its efficacy and reported a significant improvement in arthritis progression in a mouse arthritis model compared to anti-IL-17 or anti-GM-CSF monotherapy (194). These preliminary results provide a preclinical rationale for investigating combined IL-17 and GM-CSF blockade, although clinical validation is still lacking.

Methotrexate (MTX) is one of the disease-modifying antirheumatic drugs (DMARDs) used extensively to treat several autoimmune diseases and provides a long-term efficacy (195). Its promising potential increases the importance for researchers to assess its efficacy in combination with IL-17 inhibitors in patients with PsA. Patients treated with MTX combined with ixekizumab presented less American College of Rheumatology (ACR) response criteria compared to patients treated with ixekizumab monotherapy, with a similar safety profile among the two groups of patients (196). Similar results were reported in other studies (197), which suggest the ineffectiveness of MTX when combined with anti-IL-17 in treating patients with PsA. Evidence supporting the additional effect of anti-IL-17 combined with MTX remains limited, with a single case of a patient with plaque psoriasis and PsA reporting clinical improvement in skin and joint manifestations when treated with secukinumab combined with MTX, with no AEs reported (198). Larger clinical studies are needed to confirm this finding.

Phosphodiesterase (PDE) 4 is an enzyme that is responsible for the hydrolysis of second messenger cyclic adenosine monophosphate (cAMP). PDE4 inhibition with apremilast can reduce proinflammatory cytokines including IL-17, TNFα, and IFNγ. Small observational reports and case series have described variable clinical responses. The evidence remains insufficient to establish efficacy or safety of the combination. Evidence remains limited and largely observational (199–207).

### 5.2 Potential for personalized medicine in Th17-targeted therapies

Despite the major advances in managing multiple autoimmune diseases, some treatment approaches seem to be ineffective and lead to other complications because of the heterogeneity of these conditions. This highlights the need for personalized medicine to choose the optimum treatment based on the genetic, immunological, and epigenetic background of each patient in targeting Th17 cells. Several studies investigated the role of genetic biomarkers in predicting the treatment response to various immunosuppressives, including anti-TNFα and anti-IL-17, with contradictory findings.

A pharmacogenetic study has identified different SNPs associated with anti-IL17 and/or anti-IL17R treatment. Ps patients with CASP9 G/A (rs4645983), TLR5 A/G (rs5744174) and CD226 C/T (rs763361) gene polymorphisms showed a good response to anti-IL-17/anti-IL-17R treatment, while the minor alleles of CD84 G/A (rs6427528), LINC02964 G/A (rs921720) and TNF-857 C/T (rs1799724), were associated with non-response to anti-IL-17 treatment (208). In contrast, another pharmacogenetic study including 5,218 individuals from 19 clinical trials across four indications (Ps, PsA, RA, and AS) has failed to demonstrate any association between treatment response to anti-IL-17 and common variants, imputed HLA alleles, and polygenic risk scores of disease susceptibility (209).

Since the genetic biomarkers produced contradictory findings, recent research focused on clinical predictors of response to anti-IL-17 target therapy. In a real-world cohort, higher BMI was strongly associated with less favorable outcomes among patients treated with Ixekizumab (210). Constantly, lower BMI, waist circumference, and Mean body weight were associated with a higher response to Secukinumab in patients with plaque psoriasis (211). More recently, a prediction model has identified higher BMI, absence of family history of Ps, prior biologic exposure, and higher baseline PASI score as predictor parameters for non-response to IL-17 inhibitors in Ps patients (212). Additionally, in the higher response to Secukinumab group, fewer patients presented metabolic syndrome, hypertension, diabetes, previous systemic or biologic treatment, and current smoking status (211).

Immune circulating biomarkers could also provide additional information on predicting response to IL-17 inhibitors. Serum IL-22 at baseline was reported as a predictor of Disease Activity in Psoriatic Arthritis-remission (DAPSA-REM) after being exposed to anti-IL-17 treatment for one year (213). In addition, an observational study conducted on plaque psoriasis demonstrated that super responders to Secukinumab exhibited lower IL-18 and higher IL-13 at baseline, and both cytokines have been identified as independent predictors of super response, suggesting that these immune biomarkers may serve as potential biomarkers in predicting response to IL-17 inhibitors (214). However, given the small sample sizes, larger and prospective cohorts are needed to validate these biomarkers. Circulating antimicrobial peptides such as β-defensin 2 (BD-2) have been investigated to predict response to Secukinumab. In a large phase III clinical trial, patients with a higher rate of clinical responses were associated with serum BD-2 at baseline. However, BD-2 was found to be associated with adalimumab treatment, which suggests that BD-2 may not be specific only to IL-17 blockade (215).

Because of the heterogeneity of autoimmune diseases, IL-17/IL-23 molecular signature levels may differ within the affected tissues. A comparative study in PsA demonstrated that the IL-17 gene signature was higher in skin than in synovium (216), and the IL-17 cytokine family, including IL-17A, IL-17F, IL-17A/F, and IL-17C showed an elevated expression level in Ps lesional skin (217). Similarly, analysis of lesional skin and synovium tissue revealed relatively homogeneous expression of IL12B, IL23A, and IL23R in lesional skin, while their expression was heterogeneous in paired synovium (218, 219). A multi-omics study analyzed the human Ps lesion skins before and after IL-17A blockade. The treatment reduced IL-17A+ and IL-17F+ T-cells and downregulated IL-23A expression in dendritic cells, as well as several IL-17-driven inflammatory genes, including IL-36G, S100A8, DEFB4A, and DEFB4B, in suprabasal keratinocytes (220, 221). Though the studies remain limited, the IL-23/IL-17 molecular signature may contribute to future personalized medicine when selecting target therapies based on the affected tissue.

Patients with the same disease or related inflammatory diseases can differ in the immune mechanisms. T cell single profiling in Ps and RA joint patients showed that cytotoxic and regulatory T (Treg)-like tissue-resident memory T (TRM) cells were detected in both diseases, while D161+CCR6+ type 17-like TRM cells were more predominantly found in Ps joints than in RA joints (222). Similarly, immunophenotyping of peripheral blood from patients with PsA and Ps showed that CD8+CCR10+ T cells were more abundant in PsA than in Ps, and genes like RORC, IFNAR1, and ERAP1 showed higher expression in PsA than in Ps (223). Recently, paired skin and joints from patients with PsA were analyzed with single-cell RNA sequencing and spatial transcriptomics to investigate shared CD8+ T cell phenotype. The results demonstrated that Type 17 CD8+ Trm cells were abundant in both tissues, while the IL-17-related signature was stronger in the skin compared to the joint (224).

## 6 Conclusion

IL-17 blockade has been clinically validated in several autoimmune diseases and inflammatory diseases. However, its downstream mechanism does not directly modulate the broader Th17 transcriptional program or other RORγt-dependent cell population. RORγt-directed strategies provide a mechanistically broader approach by targeting Th17 differentiation and RORγt-dependent transcriptional programs, with promising results in preclinical models. Nevertheless, their clinical development remains investigational and has been limited by variable efficacy, safety and pharmacological challenges, including concerns regarding thymic effects and off-target toxicity. These translational limitations highlight the need to determine whether broader RORγt modulation can provide clinically meaningful benefits over established cytokine blockade while preserving protective immune functions.

Future development should therefore focus on improving the selectivity and tissue distribution of RORγt inhibitors, clarifying their long-term safety, and identifying patients and disease contexts most likely to benefit from different levels of Th17-axis intervention. Emerging approaches, including combination strategies, metabolic and epigenetic modulation, and biomarker-guided treatment selection, may complement direct cytokine or transcriptional targeting. A better understanding of Th17 plasticity, tissue-specific responses, and the contribution of other RORγt-expressing populations such as ILC3s will be essential for developing more precise therapeutic strategies.
